# Association between neonatal near miss and infant development: the Ribeirão Preto and São Luís birth cohorts (BRISA)

**DOI:** 10.1186/s12887-023-03897-3

**Published:** 2023-03-18

**Authors:** Liliana Yanet Gómez Aristizábal, Paulo Ricardo Higassiaraguti Rocha, Susana Cararo Confortin, Vanda Maria Ferreira Simões, Heloisa Bettiol, Marco Antonio Barbieri, Antônio Augusto Moura da Silva

**Affiliations:** 1 Federal University of Maranhão (UFMA). Graduate Program in Collective Health, Rua Barão de Itapary, 155, Maranhão 65020-070 São Luís, Brazil; 2 Ribeirão Preto Medical School, University of São Paulo – USP, Ribeirão Preto, São Paulo Brazil

**Keywords:** Child development, Neonatal near miss, Bayley scales, Birth cohort

## Abstract

**Aim:**

To analyze the association between neonatal near miss and infant development at two years.

**Methods:**

Data from two birth cohorts, one conducted in Ribeirão Preto (RP)/São Paulo and the other in São Luís (SL)/Maranhão, were used. The cognitive, motor and communication development of children was evaluated using the Bayley Scales of Infant and Toddler Development, Third Edition (Bayley-III). The following criteria were used for the definition of NNM: birth weight < 1,500 g, 5-min Apgar score < 7, gestational age < 32 weeks, and report of congenital malformations. The relationship between neonatal near miss and development was assessed using the weighted propensity score from the Inverse Probability of Treatment Weighting (IPTW). A directed acyclic graph was built to select the adjustment variables.

**Results:**

A total of 1,050 mother-newborn dyads were evaluated in SL and 1,840 in RP. Regarding outcomes in SL and RP, respectively, 2.4% and 17.3% of the children were not competent in the cognitive domain, 12.1% and 13.3% in the receptive communication domain, 39.2% and 47.1% in the expressive communication domain, 20.7% and 12.6% in the fine motor domain, and 14.3% and 13.8% in the gross motor domain. The prevalence of neonatal near miss was 5.4% in SL and 4.3% in RP. Unadjusted analysis showed an association of neonatal near miss with fine motor development in SL and RP and with the cognitive, receptive communication, expressive communication, and gross motor domains only in RP. These associations remained after adjusted analysis.

**Conclusion:**

Neonatal near miss is a risk factor for developmental delays.

**Supplementary Information:**

The online version contains supplementary material available at 10.1186/s12887-023-03897-3.

## Introduction

The growth and development of children are understood as multifactorial constructs that are part of a continuous process characterized by changes in different domains – motor, cognitive/language and psychosocial – throughout the life cycle [1, 2]. Several factors that can compromise normal development have been identified. These factors are defined as a set of biological or environmental conditions that increase the chance of developmental delays in children. The larger the number of risk factors, the greater may be the developmental impairment [3]. Conditions associated with poverty such as reduced food intake and consumption of goods and services, as well as inadequate psychosocial stimulation and unfavorable perinatal conditions, have been indicated as risk factors for development [1].

According to the literature, in the presence of risk factors such as shorter gestational age, low birth weight, and unfavorable socioeconomic condition, the likelihood of a child having motor, psychological and cognitive delays is even greater [4]. Some of these factors are part of the criteria used to define neonatal near miss (NNM), which is a set of morbid events that almost result in the death of the newborn within the first 28 days of life. Although there is no consensus on the standard definition of NNM, some pragmatic criteria such as birth weight, gestational age, 5-min Apgar score, and congenital malformations have been used for its definition [5–7]. The advantage of using this indicator is that the number of survivors who suffered from serious illnesses can be three to six times greater than the number of deaths. Based on the concept of NNM, indicators that express the newborn’s risk (severe morbidity and mortality) can also be estimated, supporting both the calculation of resources required by health services and the assessment of the quality of the provided healthcare.

Improvements in neonatal care have led to an increase in infant survival and a reduction in perinatal and neonatal mortality rates. Nevertheless, there is concern that surviving babies may have a greater risk of long-term morbidity and of exhibiting delays in different developmental domains [8]. Within this context, studies have analyzed different predictors of child development such as birth weight, premature birth, intrauterine growth restriction, and 5- and 10-min Apgar scores. Important results were obtained that revealed changes in different behavioral domains in most cases, such as motor, cognitive and language behavior [8–11].

To our knowledge, there are no studies that evaluated the infant development of children classified as NNM. Therefore, the aim of the present study was to analyze the association between NNM and infant development. Furthermore, considering that the factors associated with NNM differ between the regions of Brazil, a fact that may lead to the formation of NNM groups with different characteristics depending on geographic location, we also investigated the occurrence of NNM in two Brazilian cities located in the northeastern and southeastern regions with different socioeconomic and demographic characteristics.

## Methods

This cohort study is part of the project entitled “Etiological factors of preterm birth and consequences of perinatal factors for child health: birth cohorts in two Brazilian cities (BRISA)”. Details of the method and baseline sampling of these studies have been published previously [12] and will be explained briefly.

Two birth cohorts were started in 2010, one in Ribeirão Preto (RP)/São Paulo and the other in São Luís (SL)/Maranhão. Each cohort consisted of two components: the first was started during prenatal care and the other at birth [12]. For the present study, the population-based component at birth was used.

In RP, all infants born from January to December 2010 to mothers residing in the city were included, with 7,752 (95.7%) live births in the city that year. The 821 children who were part of the prenatal component of the study and who were born in 2010 are included in the analysis since they are also part of the birth cohort of that year. The first follow-up occurred from 2011 to 2013 and included 3,758 children in the second and third year of life [13]. All children of low or high birth weight (> 4.250 g) and one in three children of normal weight were invited to participate, which resulted in 3,758 children in the birth cohort assessed in RP from 12 to 36 months of age.

In SL, a systematic sample of 1/3 of the births that occurred in 2010 per maternity hospital was evaluated, corresponding to 5,166 (89.8%) live births. In 2013/2012, an attempt was made to follow up the entire original cohort; 3,308 children evaluated in the second year of life were followed up [13].

### Outcome variable

Infant development was evaluated in the cognitive, motor (fine and gross), and communication (expressive and receptive) domains using Bayley Scale of Infant and Toddler Development Third Edition—screening (Bayley-III screening)), validated for children aged 1 to 42 months [14]. The instrument consists of 136 questions divided into five subscales: cognitive (sensorimotor development, exploration and manipulation of familiar objects, concept formation, memory, and other cognitive aspects); receptive communication (preverbal behaviors, development of vocabulary, ability to identify objects and images); expressive communication (pre-verbal communication – such as babbling, gesturing and development of vocabulary, naming objects and images—and morphosymptomatic development – use of two words, plural, verb tense); fine motor (prehension, perceptual-motor integration, motor planning, motor speed); gross motor (dynamic movement – locomotion, coordination, balance and motor planning – and static posture).

For evaluation, the age of preterm infants was corrected by subtracting from the chronological age of follow-up the number of weeks up to the gestational age of 40 weeks. Performance on the subscales was analyzed based on the cut-off point for age established by the scale itself as Competent, Emergent and Risk. In the present study the classifications were analyzed dichotomously as competent and emergent/risk.

### Exposure variable (neonatal near miss)

The classification proposed by Silva et al. [5] was used for the definition of NNM, which considers the presence of one or more of the following conditions as a criterion: birth weight < 1,500 g, 5-min Apgar score < 7, gestational age < 32 weeks, report of congenital malformations, and use of mechanical ventilation. The last parameter was not considered in the present study because of the lack of information in the cohorts. The criteria proposed by Silva et al. [5] were validated by Kale [15] in the absence of mechanical ventilation as a pragmatic criterion and showed a good response for the classification of NNM, thus supporting the choice of the present study to use only four of the five suggested indicators.

In both cohorts, birth weight and 5-min Apgar score were collected from the registry book and medical records at the maternity hospitals. Information on newborn malformation was collected by interview with the mothers within the first 24 h after delivery. Gestational age was calculated using two criteria: the date of the last menstrual period reported by the mother or an algorithm based on the date of the last menstrual period and obstetric ultrasound when available.

### Adjustment variables

The variables were obtained using validated and standardized questionnaires applied to mothers within the first 24 h after delivery. The following adjustment variables were evaluated: gender (male and female), maternal age (< 20, 20–34, and ≥ 35 years), maternal skin color (white, brown/mulatto/cabocla/brunette, and black), maternal education level (< 8, 9 to 11, and ≥ 12 years of schooling), and socioeconomic class (A/B, C, and D/E). The last variable was evaluated according to the Brazilian Economic Classification Criteria of the Brazilian Association of Research Companies (with classes AB being the most privileged and DE the least privileged) [16]. We also evaluated the type of delivery (cesarean or normal), the number of children (continuous – 1, 2, 3, 4, 5, 6 childrens), gestational hypertension (reported by the mother as yes or no), gestational diabetes (reported by the mother as yes or no), alcohol consumption during pregnancy (yes or no), and smoking during pregnancy (yes, if at least one cigarette per day, or no).

### Data analysis

**Fig. 1:**
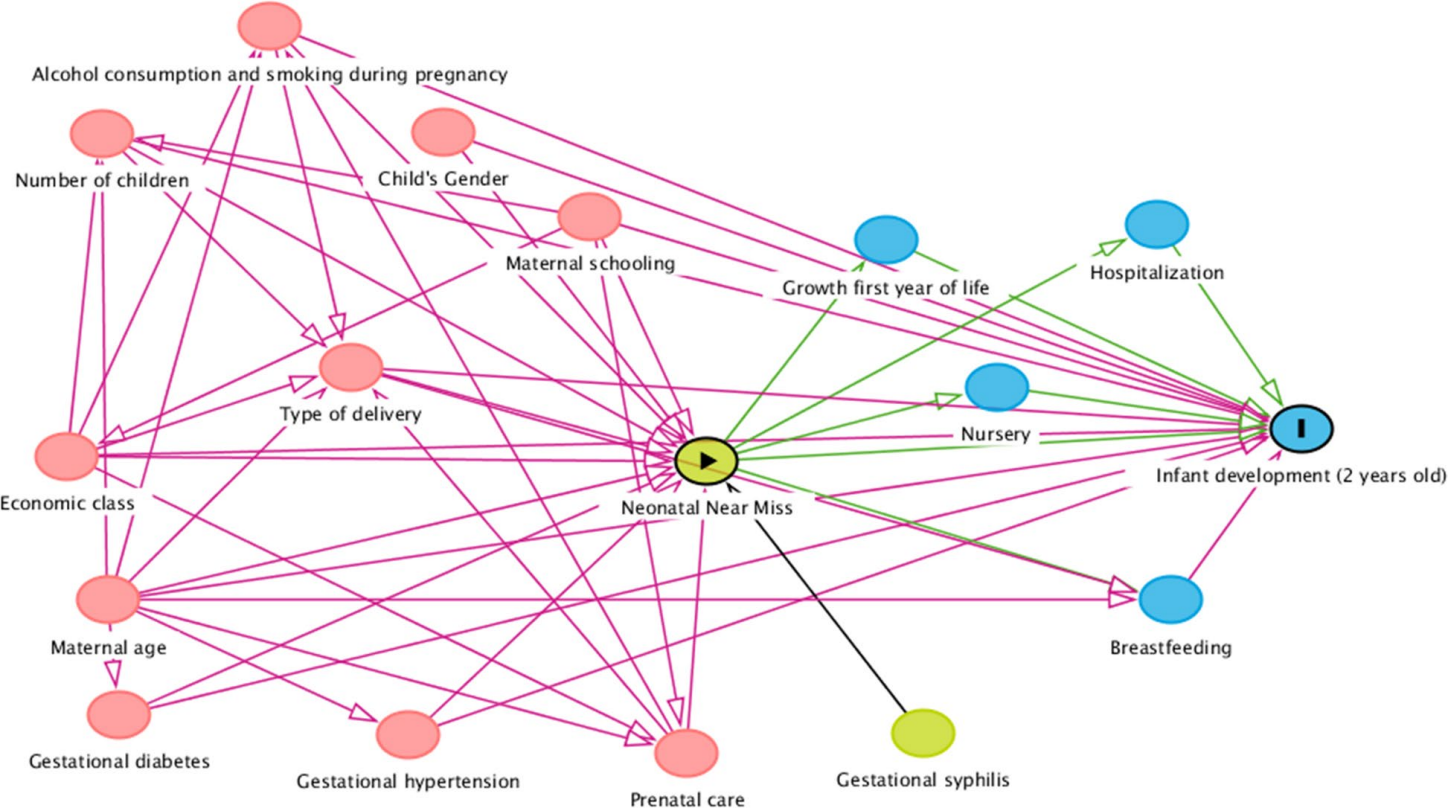
Directed acyclic graph (DAG) between near miss neonatal and infant development at two years

A directed acyclic graph was created using the DAGitty program (Fig. 1) in order to identify a minimum set of adjustment variables. DAG is a visual and qualitative tool for selecting confounding variables identified from a theoretical causal model. The arrowheads inform a causal path between two variables, and it is possible, through pre-established rules, to identify a minimum set of variables for adjustment 12. After application of DAG’s rules, the minimum adjustment set of variables for analysis were selected: child sex, maternal age, maternal education level, socioeconomic class, parity, type of delivery, gestational hypertension, gestational diabetes, and alcohol and tobacco consumption during pregnancy.

Given the occurrence of losses to follow-up, all variables were compared between children who were seen in the second follow-up and those who were not using the chi-squared test. Weighting of the sample was thus performed by calculating the probability that the child would attend the second follow-up appointment using a logistic regression model. The inverse probability was then calculated in order to minimize spurious associations resulting from sample losses and was used for weighting the model estimates. Descriptive statistics with calculation of absolute frequency, percentage, mean, and standard deviation were used.

The relationship between NNM and the developmental domains (cognitive, expressive communication, receptive communication, and fine and gross motor) was assessed using propensity scores obtained by Inverse Probability of Treatment Weighting (IPTW). Statistical analysis was performed using the R 4.0.3 program (The R Foundation for Statistical Computing).

### Ethical aspects

All procedures were approved by the Ethics Committees of the University Hospital of the Federal University of Maranhão (UFMA), São Luís (protocol number 223/2009) and of the Ribeirão Preto Medical School, University of São Paulo, Ribeirão Preto (protocol number 4.771⁄2008–30). All participants were invited and were only included in the present study after the free informed consent form was properly filled out and signed by the participant or legal representative.

## Results

A total of 1,050 mother-newborn dyads were evaluated in SL and 1,840 in RP. Regarding outcomes, 22.4% (SL) and 17.3% (RP) children were not competent in the cognitive domain, 12.1% (SL) and 13.3% (RP) were not competent in the receptive communication domain, 39.2% (SL) and 47.1% (RP) were not competent in the expressive communication domain, 20.7% (SL) and 12.6% (RP) were not competent in the fine motor domain, and 14.3% (SL) and 13.8% (RP) were not competent in the gross motor domain. The prevalence of NNM was 5.4% in SL and 4.3% in RP.

Table 1 shows the characteristics of the children. There was a predominance of boys (52.9% in SL and 50.2% in RP). The mean birth weight was 3,133.0 g (± 603.9) and 3,070.9 g (± 587.1) in SL and RP, respectively.

**Table 1:** Characterization of the study population. Ribeirão Preto and São Luís birth cohorts (BRISA), Brazil, 2010-2012

**Variable**	**São Luís**		**Ribeirão Preto**	
	**n**	**Mean **± **SD**	**n**	**Mean **± **SD**
**Maternal age (years)**		25.2 ± 6.1		27.0 ± 6. 2
**Gestational age (weeks)**		38.0 ± 2.8		38.4 ± 2.4
**Birth weight (g)**		3,133.0 ± 603.9		3,070.9 ± 587.1
**Child age (months)**		17.44 ± 3.65		20.62 ± 4.42
**Number of children**		1.7 ± 1.0		-
	**n**	**%**	**n**	**%**
**Neonatal near miss**				
Yes	57	5.4	79	4.3
No	993	94.6	1,761	95.7
**Low birth weight**				
Yes	15	1.4	36	2.0
No	1,035	98.6	1,804	98.0
**Gestational age <32 weeks**				
Yes	32	3.1	43	2.3
No	1,018	97.0	1,797	97.7
**Apgar score <5**				
Yes	15	1.4	15	0.8
No	1,035	98.6	1,822	99.2
**Congenital malformation**				
Yes	7	0.7	21	1.1
No	1,043	99.3	1,819	98.9
**Child sex**				
Female	495	47.1	917	49.8
Male	555	52.9	923	50.2
**Type of delivery**				
Cesarean	495	47.1	1,033	56.1
Normal	555	52.9	807	43.9
**Cognitive**				
Competent	815	77.6	1,544	82.7
Not competent	235	22.4	322	17.3
**Receptive communication**				
Competent	923	87.9	1,617	86.7
Not competent	127	12.1	249	13.3
**Expressive communication**				
Competent	638	60.8	987	52.9
Not competent	412	39.2	879	47.1
**Fine motor**				
Competent	233	79.3	1,631	87.4
Not competent	217	20.7	235	12.6
**Gross motor**				
Competent	908	85.7	1,609	86.2
Not competent	152	14.3	257	13.8
**Maternal skin color**				
White	178	17.0	1,059	57.6
Brown/mulatto/cabocla/brunette	710	67.6	587	31.9
Black	162	15.4	194	10.5
**Maternal educational level**				
<8 years	237	22.6	405	22.0
9 to 11 years	671	63.9	1,092	59.4
>12 years	142	13.5	343	18.6
**Maternal age**				
<20 years	206	19.6	223	12.1
20 to 34 years	739	70.4	1,331	72.3
≥35 years	105	10.0	286	1.5
**Socioeconomic class**				
A/B	154	14.7	749	42.9
C	629	59.9	868	49.7
D/E	267	25.4	129	7.4
**Gestational hypertension**				
Yes	186	17.7	265	14.4
No	864	82.3	1,575	85.6
**Gestational diabetes**				
Yes	28	2.6	116	6.3
No	1,032	97.4	1,724	93.7
**Alcohol use during pregnancy**				
Yes	141	13.4	443	24.1
No	909	86.6	1,397	75.9
**Smoking during pregnancy**				
Yes	29	2.8	213	11.6
No	1,021	97.2	1,627	88.4

With respect to maternal characteristics, 67.6% of mothers in SL had a brown/mulatto/cabocla/brunette skin color and 57.6% of mothers in RP were white. Most mothers had 9 to 11 years of schooling (63.9% in SL and 59.4% in RP), ranged in age from 20 to 34 years (70.4% in SL and 72.3% in RP), and belonged to socioeconomic class C (59.9% in SL and 49.7% in RP). Regarding the type of delivery, 47.1% (SL) and 56.1% (RP) had a cesarean section. Gestational hypertension was observed in 17.7% of women in SL and 14.4% in RP. Only 2.6% and 6.3% of mothers in SL and RP had gestational diabetes, respectively. The prevalence of alcohol consumption during pregnancy was 13.4% in SL and 24.1% in RP and that of smoking during pregnancy was 2.8% in SL and 11.6% in RP. The mean maternal age was 25.2 years (± 6.1) and 27.0 years (± 6.2) in SL and RP, respectively; the mean gestational age was 38.0 months (± 2.8) and 38.4 (± 2.4) months and the mean number of children was 1.7 (± 1.0).

Table 2 shows the results of unadjusted and adjusted analyses of the association between NNM and the outcomes. In unadjusted analysis, an association was observed between NNM and fine motor development in SL and RP. Neonatal near miss was associated with the cognitive, receptive communication, expressive communication, and gross motor domains only in RP. These associations remained in the adjusted analysis. Children with NNM from SL and RP were, respectively, 1.87 (OR: 1.87; 95%CI: 1.01 – 3.46) and 2.74 (OR: 2.74; 95%CI: 1.47 – 5.12) times more likely to be not competent in the fine motor domain when compared to competent children.

**Table 2:** Effect of neonatal near miss on infant development at two years. Ribeirão Preto and São Luís birth cohorts (BRISA), Brazil, 2010–2012

	**São Luís**		**Ribeirão Preto**	
	**Unadjusted analysis**	Adjusted analysis*	**Unadjusted analysis**	Adjusted analysis*
	**OR (95%CI)**	**OR (95%CI)**	**OR (95%CI)**	**OR (95%CI)**
**Cognitive**				
**Near miss**				
No	1	1	1	1
Yes	1.70 (0.94—3.09)	1.71 (0.92—3.20)	3.94 ( 2.48—6.27)	3.00 (1.68—5.35)
**Receptive communication**				
**Near miss**				
No	1	1	1	1
Yes	1.09 (0.50—2.40)	1.40 (0.62—3.17)	3.59 (2.21—5.85)	3.10 (1.69—5.69)
**Expressive communication**				
**Near miss**				
No	1	1	1	1
Yes	1.20 (0.68—2.06)	1.40 (0.64—2.05)	2.37 (1.47—3.83)	3.10 (1.53—4.91)
**Gross motor**				
**Near miss**				
No	1	1	1	1
Yes	1.47 (0.75—2.87)	1.48 (0.72—3.03)	4.74 (2.96—7.59)	5.43 (3.04—9.69)
**Fine motor**				
**Near miss**				
No	1	1	1	1
Yes	1.99 (1.11—3.57)	1.87 (1.01—3.46)	3.01 (1.81—5.00)	2.74 (1.47- 5.12)

^*^Adjusted for socioeconomic class, maternal education level, maternal age, gestational hypertension, gestational diabetes, parity, child sex, type of delivery, alcohol use during pregnancy, and smoking during pregnancy

Regarding the other domains that were associated with NNM only RP, children with the condition were approximately 3 times more likely to be not competent in the cognitive (OR: 3.00; 95%CI: 1.68 – 5.35), receptive communication (OR: 3.10; 95%CI: 1.69 – 5.69) and expressive communication (OR: 3.10; 95%CI: 1.53 – 4.91) domains, and 5 times more likely to be not competent in the gross motor domain (OR: 5.43; 95%CI: 3.04 – 96.9) when compared to their peers (Table 2).

## Discussion

In RP, the NNM group exhibited delays in all development scales compared to the non-NNM group. On the other hand, in SL, children in the NNM group showed worse performance than their peers only in fine motor development. These results suggest that the association of NNM morbidity with developmental delays in childhood may vary depending on the characteristics of the studied location.

The association between NNM and low performance in the developmental indicators observed in RP reinforces evidence showing significant delays in the development of very low birth weight (< 1,500 g) and preterm children (gestational age < 32 weeks) [17], as well as children with a 5-min Apgar score < 7 [10] and with congenital malformations [18]. Theanatomical alterations observed in children born with these characteristics, such as reduced whole brain volume [19], insular and temporal lobe and gray matter reductions [20], morphological alterations accompanied by reduced gray and white matter complexity [21], hippocampal, thalamus and cerebellar volume reductions [21–23], and altered functional connectivity in the brain [24], have been associated with behavioral delays. Nevertheless, deficits in the development of children with NNM cannot be explained solely by these structural alterations since stressful events experienced by this population early in life, for example, being more frequently submitted to medical interventions, can affect the organization of the central nervous system and can cause important physiological and behavioral changes in the child [25].

The discrepancy in the association between NNM and development observed between RP and SL can be explained in part by differences in the characteristics of the NNM groups between the two cities. Although the present results corroborate those of previous studies that did not find disparities in the frequency of NNM between different regions of Brazil [5], the factors associated with the occurrence of NNM can vary according to region [6, 26]. Rocha et al. (2022) showed that a low maternal educational level, living without a companion, gestational hypertension, smoking during pregnancy, and cesarean delivery were associated with NNM in RP but not in SL [27]. Thus, the particularities of each location tend to lead to the formation of NNM groups with different characteristics, a fact that would explain different outcomes in the developmental domains.

Exposure to alcohol and/or tobacco during pregnancy [28–30], cesarean delivery [27], high blood pressure [31], and gestational diabetes [32] are generally associated with long-term developmental delays [28]. Furthermore, cesarean delivery has been associated with changes in the intestinal microbiota, which plays an important role in child development and behavior [33–35].

In SL, an association between NNM and developmental delays was only observed for fine motor skills. These data suggest that, despite the adverse conditions experienced during the fetal period and at birth, children in the NNM group are able to recover and achieve expected patterns of typical development in the second year. On the other hand, the occurrence of typical behaviors within the expected time window in NNM children may have been due to the non-linearity and temporal variability that frequently characterize the development of children exposed to perinatal risk factors [21, 36]. However, it should be noted that, for all domains evaluated in the present study, the percentage of children classified as at risk of not being competent was higher in the NNM group. These results highlight the importance of following up these children over time, as studies have reported an increase in the difficulties of children born with adverse conditions, especially at school age.

Some limitations of this study must be mentioned, such as the use of the shortened version of the Bayley Scales for assessing infant development. However, this version allows to screen for possible developmental delays in studies involving a large number of participants. Another limitation is the difference in the mean age at which the children were evaluated in the two cities. However, in order to minimize the effect of age, we decided to use age-specific cut-off points for classification.

As strengths of this study, we mention the fact that the two birth cohorts were started in the same year and were followed up in two cities with contrasting socioeconomic and demographic characteristics. In addition, we highlight the originality of the present study that evaluated the development of NNM children in cohorts conducted in cities located in different regions of Brazil using the same methodology. Largen numbers in study is also stregth of the study.

In conclusion, NNM was associated with cognitive, motor and language delays in the second year of life in RP. However, in SL, a difference between the NNM and non-NNM groups was only observed for fine motor skills. These results suggest that, although the criteria used for the definition of NNM are known risk factors for child development, the characteristics of the groups resulting from the social and cultural differences between the cities studied seem to influence the relationship between NNM and infant development in the second year of life.

## Supplementary Information

**Additional file 1.**

## Data Availability

The data that support the findings of this study are available from e-mail rosangela.flb@ufma.br and hbettiol@fmrp.usp.br, but restrictions apply to the availability of these data, which were used under license for the current study, and so are not publicly available. Data are however available from the authors upon reasonable request and with permission of Rosangela Fernandes Lucena Batista and Heloisa Bettiol.
