# Supplementary material for: Association between neonatal near miss and infant development: the Ribeirão Preto and São Luís birth cohorts (BRISA)
Source: BMC Pediatr. 2023 Mar 18;23:125. doi: 10.1186/s12887-023-03897-3 (PMC10024445; doi:10.1186/s12887-023-03897-3)
Supplement: Supplementary file 1 — Additional file 1. [file 12887_2023_3897_MOESM1_ESM.docx]

**SUPPLEMENTARY MATERIAL**

Characterization of the study population by Neonatal Near miss group. Ribeirão Preto and São Luís birth cohorts (BRISA), Brazil, 2010-2012.

|  | **São Luís** | | | |  | **Reibeirão Preto** | | | |
| --- | --- | --- | --- | --- | --- | --- | --- | --- | --- |
|  | **Neonatal Near Miss** | | | |  | **Neonatal Near Miss** | | | |
|  | **Yes** | | **No** | |  | **Yes** | | **No** | |
| **Variable** | **n** | **%** | **n** | **%** |  | **n** | **%** | **n** | **%** |
| **Low birth weight** |  |  |  |  |  |  |  |  |  |
| Yes | 15 | 4,0 | 0 | 0 |  | 36 | 1,96 | 0 | 0 |
| No | 42 | 1,43 | 993 | 94,57 |  | 43 | 2,34 | 1761 | 95,71 |
| **Gestational age <32 weeks** |  |  |  |  |  |  |  |  |  |
| Yes | 32 | 3,05 | 0 | 0 |  | 43 | 2,34 | 0 | 0 |
| No | 25 | 2,38 | 993 | 94,57 |  | 36 | 1,96 | 1761 | 95,71 |
| **Apgar score <5** |  |  |  |  |  |  |  |  |  |
| Yes | 15 | 1,43 | 0 | 0 |  | 15 | 0,82 | 0 | 0 |
| No | 42 | 4,00 | 993 | 94,57 |  | 61 | 3,32 | 1761 | 95,71 |
| **Congenital malformation** |  |  |  |  |  |  |  |  |  |
| Yes | 7 | 0,67 | 0 | 0 |  | 17 | 0,92 | 4 | 0,22 |
| No | 50 | 4,76 | 993 | 94,57 |  | 62 | 3,37 | 1757 | 95,49 |
| **Child sex** |  |  |  |  |  |  |  |  |  |
| Female | 24 | 2,29 | 471 | 44,86 |  | 38 | 2,07 | 879 | 47,77 |
| Male | 33 | 3,14 | 522 | 49,71 |  | 41 | 2,23 | 882 | 47,93 |
| **Type of delivery** |  |  |  |  |  |  |  |  |  |
| Cesarean | 19 | 1,81 | 476 | 45,33 |  | 52 | 2,83 | 981 | 53,32 |
| Normal | 38 | 3,62 | 516 | 49,24 |  | 27 | 1,47 | 780 | 42,39 |
| **Cognitive** |  |  |  |  |  |  |  |  |  |
| Competent | 39 | 3,71 | 776 | 73,9 |  | 45 | 2,45 | 1478 | 80,33 |
| Not competent | 18 | 1,71 | 217 | 20,67 |  | 34 | 1,85 | 283 | 15,38 |
| **Receptive communication** |  |  |  |  |  |  |  |  |  |
| Competent | 49 | 4,67 | 874 | 83,24 |  | 52 | 2,83 | 1539 | 83,64 |
| Not competent | 8 | 0,76 | 119 | 11,33 |  | 27 | 1,47 | 222 | 12,07 |
| **Expressive communication** |  |  |  |  |  |  |  |  |  |
| Competent | 32 | 3,05 | 606 | 57,71 |  | 26 | 1,41 | 948 | 51,52 |
| Not competent | 25 | 2,38 | 387 | 36,86 |  | 53 | 2,88 | 813 | 44,18 |
| **Fine motor** |  |  |  |  |  |  |  |  |  |
| Competent | 38 | 4,29 | 795 | 75,71 |  | 56 | 3,04 | 1550 | 84,24 |
| Not competent | 19 | 1,14 | 198 | 18,86 |  | 23 | 1,25 | 211 | 11,47 |
| **Gross motor** |  |  |  |  |  |  |  |  |  |
| Competent | 45 | 3,62 | 853 | 81,24 |  | 47 | 2,55 | 1540 | 83,7 |
| Not competent | 12 | 1,81 | 140 | 13,33 |  | 32 | 1,74 | 221 | 12,01 |
| **Maternal skin color** |  |  |  |  |  |  |  |  |  |
| White | 9 | 0,86 | 169 | 16,10 |  | 37 | 2,01 | 1022 | 55,54 |
| Brown/mulatto/cabocla/brunette | 38 | 3,62 | 672 | 64,00 |  | 28 | 1,52 | 559 | 30,38 |
| Black | 10 | 0,95 | 152 | 14,48 |  | 14 | 0,76 | 180 | 9,78 |
| **Maternal educational level** |  |  |  |  |  |  |  |  |  |
| <8 years | 17 | 1,62 | 220 | 20,96 |  | 25 | 1,41 | 379 | 20,6 |
| 9 to 11 years | 31 | 2,95 | 640 | 60,95 |  | 43 | 2,34 | 1049 | 57,01 |
| >12 years | 9 | 0,86 | 133 | 12,67 |  | 10 | 0,54 | 333 | 18,1 |
| **Maternal age** |  |  |  |  |  |  |  |  |  |
| <20 years | 14 | 1,33 | 192 | 18,29 |  | 14 | 0,76 | 209 | 11,36 |
| 20 to 34 years | 37 | 3,52 | 702 | 9,43 |  | 55 | 2,99 | 1276 | 69,35 |
| ≥35 years | 6 | 0,57 | 99 | 66,86 |  | 10 | 0,54 | 276 | 15 |
| **Socioeconomic class** |  |  |  |  |  |  |  |  |  |
| A/B | 8 | 0,76 | 146 | 13,9 |  | 29 | 1,66 | 720 | 41,24 |
| C | 35 | 3,33 | 594 | 56,57 |  | 33 | 1,89 | 835 | 47,82 |
| D/E | 14 | 1,33 | 253 | 24,1 |  | 11 | 0,63 | 118 | 6,76 |
| **Gestational hypertension** |  |  |  |  |  |  |  |  |  |
| Yes | 13 | 1,24 | 173 | 16,48 |  | 21 | 3,15 | 244 | 13,26 |
| No | 44 | 4,19 | 820 | 78,1 |  | 58 | 1,14 | 1517 | 82,45 |
| **Gestational diabetes** |  |  |  |  |  |  |  |  |  |
| Yes | 1 | 0,1 | 26 | 2,48 |  | 3 | 0,16 | 113 | 6,14 |
| No | 56 | 5,33 | 967 | 92,1 |  | 76 | 4,13 | 1648 | 89,57 |
| **Alcohol use during pregnancy** |  |  |  |  |  |  |  |  |  |
| Yes | 6 | 0,57 | 135 | 12,86 |  | 33 | 2,5 | 410 | 22,28 |
| No | 51 | 4,86 | 858 | 81,71 |  | 46 | 1,79 | 1351 | 73,42 |
| **Smoking during pregnancy** |  |  |  |  |  |  |  |  |  |
| Yes | 1 | 5,33 | 28 | 2,67 |  | 15 | 3,48 | 198 | 10,76 |
| No | 56 | 0,1 | 965 | 91,9 |  | 64 | 0,82 | 1563 | 84,95 |
